# Crystal and mol­ecular structure of (2*Z*,5*Z*)-3-(2-meth­oxy­phen­yl)-2-[(2-meth­oxy­phen­yl)imino]-5-(4-nitro­benzyl­idene)thia­zolidin-4-one

**DOI:** 10.1107/S2056989017003218

**Published:** 2017-03-14

**Authors:** Ahmed Djafri, Abdelkader Chouaih, Jean-Claude Daran, Ayada Djafri, Fodil Hamzaoui

**Affiliations:** a Laboratory of Technology and Solid Properties (LTPS), Abdelhamid Ibn Badis University, BP 227 Mostaganem 27000, Algeria; b Centre de Recherche Scientifique et Technique en Analyses Physico-chimiques (CRAPC), BP 384-Bou-Ismail-RP 42004, Tipaza, Algeria; c Laboratoire de Chimie de Coordination, UPR-CNRS 8241, 205, route de Narbonne, 31077 Toulouse Cedex, France; d Laboratory of Organic Applied Synthesis (LSOA), Department of Chemistry, Faculty of Sciences, University of Oran 1, Ahmed Ben Bella, 31000 Oran, Algeria

**Keywords:** crystal structure, thia­zolidin-4-one, DFT calculations, hydrogen bonding, π–π inter­actions

## Abstract

In the title compound, C_24_H_19_N_3_O_5_S, the thia­zole ring (r.m.s. deviation = 0.012 Å) displays a planar geometry and is surrounded by three fragments, two meth­oxy­phenyl and one nitro­phenyl. The thia­zole ring is almost in the same plane as the nitro­phenyl ring, making a dihedral angle of 20.92 (6)°. The two meth­oxy­phenyl groups are perpendicular to the thia­zole ring [dihedral angles of 79.29 (6) and 71.31 (7)° and make a dihedral angle of 68.59 (7)°. The mol­ecule exists in an *Z*,*Z* conformation with respect to the C=N imine bond. In the crystal, a series of C—H⋯N, C—H⋯O and C—H⋯S hydrogen bonds, augmented by several π–π(ring) inter­actions, produce a three-dimensional architecture of mol­ecules stacked along the *b*-axis direction. The experimentally derived structure is compered with that calculated theoretically using DFT(B3YLP) methods.

## Abstract 2

In the title mol­ecule, both meth­oxy­phenyl groups are nearly perpendicular to the thia­zole ring and are nearly perpendicular to each other. In the crystal, a series of C—H⋯N, C—H⋯O and C—H⋯S hydrogen bonds, augmented by several C—H⋯π(ring) inter­actions, produce a three-dimensional architecture of mol­ecules stacked along the *b*-axis direction.

## Chemical context   

There are numerous studies of simple thia­zoles reporting their biological activity (Saeed *et al.*, 2010 ▸; Shokol *et al.*, 2013 ▸; Akhtar *et al.*, 2007 ▸). As a result of their properties, thia­zole derivatives are inter­esting candidates for obtaining new materials. Thia­zole compounds have been also studied for their non-linear optical properties (Smokal *et al.*, 2009 ▸). Recently, numerous studies have reported the theoretical and experimental structures of this kind of compound (Boulakoud *et al.*, 2015 ▸; Khelloul *et al.*, 2016 ▸). Prompted by these investigations and in a continuation of our research on the development of organic heterocyclic compounds (Toubal *et al.*, 2012 ▸; Rahmani *et al.*, 2016 ▸; Bahoussi *et al.*, 2017 ▸), we report in this paper the synthesis and crystal structure of the compound (2*Z*,5*Z*)-5-(4-nitro­benzyl­idene)-3-(2-meth­oxy­phen­yl)-2-[(2-meth­oxy­phenyl)imino]­thia­zolidin-4-one. The experimental geometric parameters are compared with those optimized by density functional theory (DFT).

## Structural commentary   

**Figure 1:**
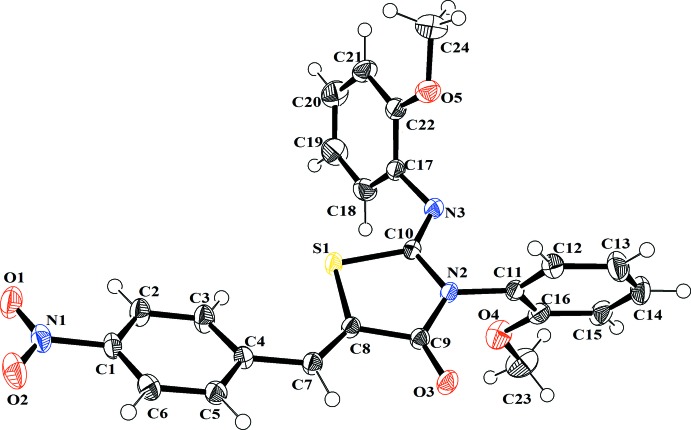
Crystal structure of the title compound, with the atom-numbering scheme (displacement ellipsoids are drawn at the 50% probability level). H atoms are shown as small spheres of arbitrary radii.

The mol­ecular structure of the title compound with the atomic numbering scheme is shown in Fig. 1 ▸. All of the bond lengths are within normal ranges. Bond lengths and angles for the 5-(4-nitro­benzyl­idene)-3-(2-meth­oxy­phen­yl) moiety are consistent with those in related structures (Benhalima *et al.*, 2011 ▸). As always, the thiazole ring is close to planar (r.m.s. deviation = 0.012 Å) and is surrounded by three fragments, two meth­oxy­phenyl and nitro­phenyl. The central thia­zole ring is twisted by −2.9 (2)° (C4—C7—C8—S1) to the nitro­phenyl ring, by −71.58 (18) (C10—N3—C17—C18) to the first meth­oxy­phenyl group and by −80.62 (15)° (C10—N2—C11—C16) to the second meth­oxy­phenyl group. The dihedral angles between the thia­zole ring and these three phenyl rings are 20.92 (6), 79.29 (6) and 71.31 (7)°, respectively. The mol­ecule exists in an *Z*,*Z* conformation with respect to the C10=N3 imine bond. Some bond angles of the aromatic rings are slightly out of normal range due to the presence of the meth­oxy and nitro substituents, *viz.* C4—C5 = 1.4040 (17), C12—C11 = 1.3724 (19), C22—C17 = 1.4046 (19) Å; C2—C1—C6 = 122.26 (12), C3—C4—C5 = 118.42 (12), C12—C13—C14 = 118.78 (14), C13—C14—C15 = 121.52 (14), C19—C20—C21 = 121.28 (14)°.
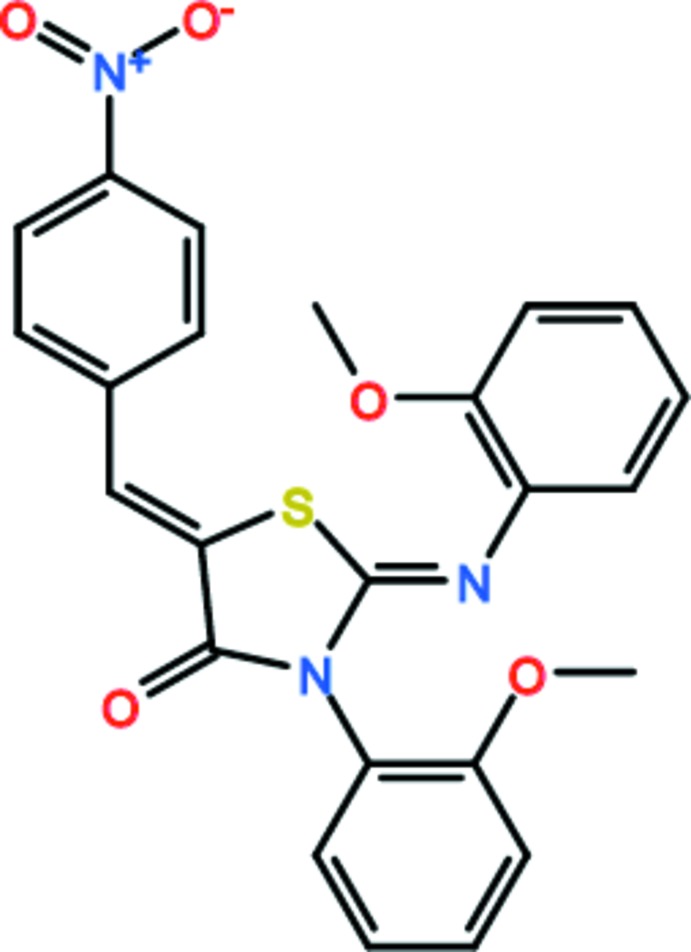


## Supra­molecular features   

**Figure 2:**
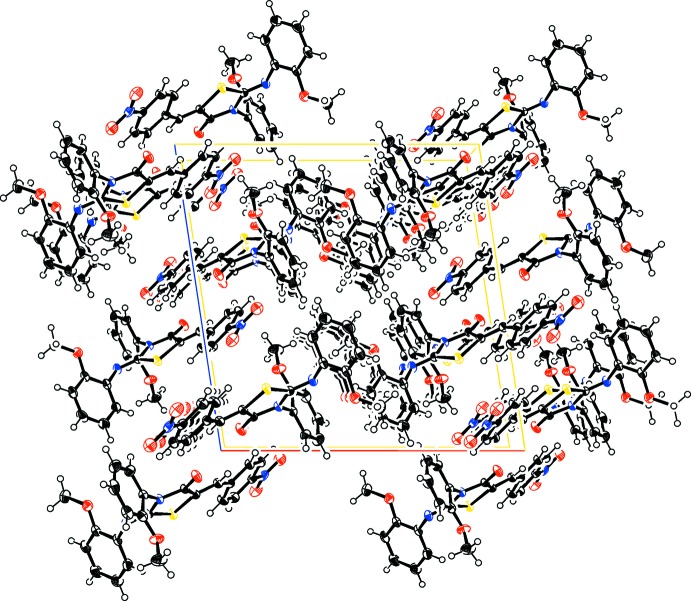
The crystal packing viewed along the *c* axis.

In the extended structure of the title compound, weak C—H⋯N, C—H⋯O and C—H⋯S hydrogen bonds (Table 1 ▸, Fig. 2 ▸) connect the mol­ecules into a three-dimensional supra­molecular network. π–π stacking involving the benzene rings is also observed [*Cg*⋯*Cg*(−*x*, −*y*, −*z*) = 3.7664 (8) Å; *Cg* is the centroid of the C1–C6 ring].

## Quantum-chemical calculations   

**Figure 3:**
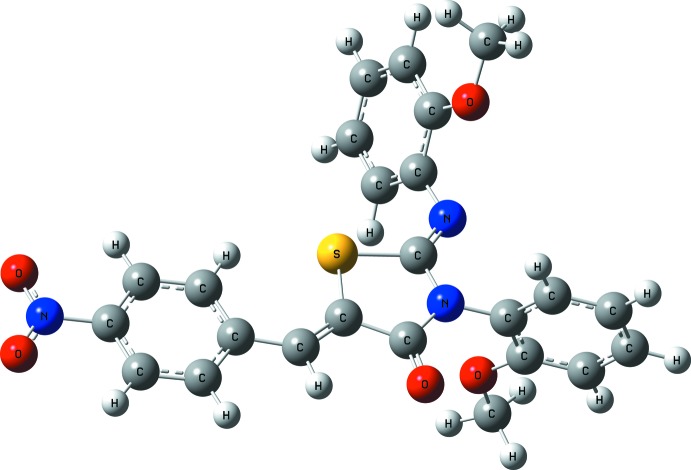
Optimized structure of the title compound, calculated at the B3LYP/6–31 G(d,p) level.

Geometry optimization has been performed using DFT(B3YLP) methods with the 6-31G(d,p) basis set (Becke, 1997 ▸; Rauhut & Pulay, 1995 ▸). All calculations were carried out by using *Gaussian* package (Frisch *et al.*, 2004 ▸) and the obtained data visualized by means of *GaussView 4.1* (Dennington *et al.*, 2007 ▸). The optimized structure is shown in Fig. 3 ▸. The calculated geometrical parameters such as bond lengths, bond angles and torsion angles (given in the Supporting information) are in good agreement with experimental values on basis of the diffraction study. The torsion angle between the first meth­oxy­phenyl ring and the thia­zole ring is −67.40° [experimental: −71.58 (18)°] and between the second meth­oxy­phenyl ring and the thia­zole ring is −84.61° [experimental: −80.62 (15)°].

## Synthesis and crystallization   

**Figure 4:**
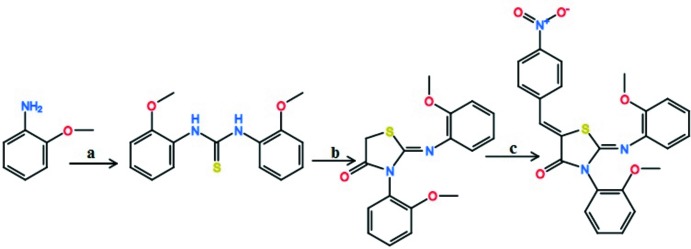
Chemical pathways showing the formation of the title compound. Reagents and conditions: (*a*) CS_2_, EtOH, 346 K; (*b*) BrAcOEt, EtOH, CH_3_COONa 348 K; (*c*) NO_2_C_6_H_4_CHO; CH_3_COOH; CH_3_COONa, 365 K.

The synthesis of the title compound was performed according to the scheme in Fig. 4 ▸. To a solution of *o*-anisidine (0.02 mol) in ethanol (10 mL) was added carbon di­sulfide (0.01 mol) and the resulting solution was refluxed for 6 h to gave *N*,*N*′ diaryl thio­uria. (0.01 mol) of the compound and (0.01 mol) of ethyl bromo­acetate were refluxed in 40 mL of absolute ethanol in the presence of (0.04 mol) of anhydrous CH_3_COONa for 2 h. The precipitate thus obtained was filtered, dried and recrystallized from ethanol to formed 3-*N*-(2-meth­oxy­phen­yl)-2-*N*′-(2-meth­oxy­phenyl­imino)-thia­zolidin-4-one. 4-Nitro­benz­alde­hyde (0.01 mol) was added to a solution of the latter compound in 10 mL of acetic acid containing three equivalents of anhydrous sodium acetate. The reaction mixture was refluxed for 4 h and monitored by TLC on silica gel using di­chloro­methane:ethyl acetate (9:1) as a solvent system. The separated solid was filtered, washed with cold water and dried to give the title compound. Single crystals suitable for X-ray diffraction were obtained from ethanol solution.

Spectroscopic data (FT–IR, ^1^H NMR and ^13^C NMR). IR (KBr, cm^−1^): 2941 (C—H), 1723 (C=O), 1516 (C=N), 1023 (C—N), 751 (C—S). ^1^H NMR, (CDCl_3_, 300 MHz) δ (ppm) *J* (Hz): 3.72 (*s*, 3H, OCH_3_), 3.82 (*s*, 3H, OCH_3_), 6.83 (*m*, 3H, Ar-H), 7.06 (*m*, 3H, Ar-H), 7.36–7.06 (*m*, 3H, Ar-H), 7.54 (*d*, 2H, *J* = 8.81 Hz, Ar-H), 7.73 (*s*, 1H, C=CH), 8.18 (*d*, 2H, *J* = 8.81 Hz, Ar-H). ^13^C NMR, (CDCl_3_, 300 MHz) δ (ppm): 55.90 (OCH_3_), 55.98 (OCH_3_), 112.24, 112.59, 120.99, 121.21, 121.85, 123.15, 124.17, 126.07, 126.93, 127.44, 129.85, 130.38, 131.12, 137.33, 140.12, 147.46, 150.09, 150.65, 155.02, 165.69 (C=O).

## Refinement   

Crystal data, data collection and structure refinement details are summarized in Table 2 ▸. H atoms were placed in calculated positions (C—H = 0.96–1.08 Å) and refined using a riding mode with *U*_iso_(H) = 1.5*U*_eq_(C) for methyl H atoms and 1.2*U*_eq_(C) for other H atoms.

## Supplementary Material

Crystal structure: contains datablock(s) I, global. DOI: 10.1107/S2056989017003218/xu5900sup1.cif

Structure factors: contains datablock(s) I. DOI: 10.1107/S2056989017003218/xu5900Isup2.hkl

Supporting information file. DOI: 10.1107/S2056989017003218/xu5900Isup5.cml

Calculated geometric parameters. DOI: 10.1107/S2056989017003218/xu5900sup3.docx

Geometrical parameters calculated theoretically. DOI: 10.1107/S2056989017003218/xu5900sup3.pdf

CCDC reference: 1534261

Additional supporting information:  crystallographic information; 3D view; checkCIF report

## Figures and Tables

**Table 1:** Hydrogen-bond geometry (Å, °)

*D*—H⋯*A*	*D*—H	H⋯*A*	*D*⋯*A*	*D*—H⋯*A*
C3—H3⋯S1	0.95	2.58	3.2594 (14)	128
C3—H3⋯O1^i^	0.95	2.57	3.3320 (18)	138
C5—H5⋯O3^ii^	0.95	2.58	3.3938 (17)	145
C7—H7⋯O3^ii^	0.95	2.40	3.1982 (15)	142
C21—H21⋯N3^iii^	0.95	2.52	3.4576 (19)	170
C23—H23*C*⋯O1^iv^	0.98	2.55	3.238 (2)	127

Symmetry codes: (i) 
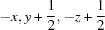
; (ii) 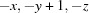; (iii) 
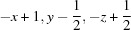
; (iv) 
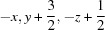
.

**Table 2:** Experimental details

Crystal data	
Chemical formula	C_24_H_19_N_3_O_5_S
*M*_r_	461.48
Crystal system, space group	Monoclinic, *P*2_1_/*c*
Temperature (K)	173
*a*, *b*, *c* (Å)	15.6096 (4), 8.8817 (2), 15.8973 (4)
β (°)	98.601 (2)
*V* (Å^3^)	2179.21 (9)
*Z*	4
Radiation type	Mo *K*α
μ (mm^−1^)	0.19
Crystal size (mm)	0.58 × 0.21 × 0.20

Data collection	
Diffractometer	Nonius Kappa CCD
Absorption correction	ψ scan (North *et al.*, 1968 ▸)
*T*_min_, *T*_max_	0.856, 0.919
No. of measured, independent and observed [*I* > 2σ(*I*)] reflections	29723, 6435, 5119
*R*_int_	0.031
(sin θ/λ)_max_ (Å^−1^)	0.727

Refinement	
*R*[*F* ^2^ > 2σ(*F* ^2^)], *wR*(*F* ^2^), *S*	0.041, 0.107, 1.03
No. of reflections	6435
No. of parameters	300
H-atom treatment	H-atom parameters constrained
Δρ_max_, Δρ_min_ (e Å^−3^)	0.43, −0.29

Computer programs: KappaCCD (Nonius, 1998 ▸), *DENZO* and *SCALEPACK* (Otwinowski & Minor, 1997 ▸), *SHELXS97* (Sheldrick, 2008 ▸), *SHELXL2014* (Sheldrick, 2015 ▸), *ORTEP-3 for Windows* (Farrugia, 2012 ▸) and *Mercury* (Macrae *et al.*, 2006 ▸).

## supplementary crystallographic information

### Crystal data

**Table:**

C_24_H_19_N_3_O_5_S	*F*(000) = 960
*M**_r_* = 461.48	*D*_x_ = 1.407 Mg m^−^^3^
Monoclinic, *P*2_1_/*c*	Mo *K*α radiation, λ = 0.71073 Å
*a* = 15.6096 (4) Å	Cell parameters from 100 reflections
*b* = 8.8817 (2) Å	θ = 2–29°
*c* = 15.8973 (4) Å	µ = 0.19 mm^−^^1^
β = 98.601 (2)°	*T* = 173 K
*V* = 2179.21 (9) Å^3^	Prism, colourless
*Z* = 4	0.58 × 0.21 × 0.20 mm

### Data collection

**Table:**

Nonius Kappa CCD diffractometer	5119 reflections with *I* > 2σ(*I*)
θ/2θ scans	*R*_int_ = 0.031
Absorption correction: ψ scan (North *et al.*, 1968)	θ_max_ = 31.1°, θ_min_ = 3.0°
*T*_min_ = 0.856, *T*_max_ = 0.919	*h* = −22→22
29723 measured reflections	*k* = −12→11
6435 independent reflections	*l* = −21→22

### Refinement

**Table:**

Refinement on *F*^2^	0 restraints
Least-squares matrix: full	Hydrogen site location: inferred from neighbouring sites
*R*[*F*^2^ > 2σ(*F*^2^)] = 0.041	H-atom parameters constrained
*wR*(*F*^2^) = 0.107	*w* = 1/[σ^2^(*F*_o_^2^) + (0.0467*P*)^2^ + 0.9373*P*] where *P* = (*F*_o_^2^ + 2*F*_c_^2^)/3
*S* = 1.03	(Δ/σ)_max_ < 0.001
6435 reflections	Δρ_max_ = 0.43 e Å^−^^3^
300 parameters	Δρ_min_ = −0.29 e Å^−^^3^

### Special details

**Table:**

Geometry. All esds (except the esd in the dihedral angle between two l.s. planes) are estimated using the full covariance matrix. The cell esds are taken into account individually in the estimation of esds in distances, angles and torsion angles; correlations between esds in cell parameters are only used when they are defined by crystal symmetry. An approximate (isotropic) treatment of cell esds is used for estimating esds involving l.s. planes.

### Fractional atomic coordinates and isotropic or equivalent isotropic displacement parameters (Å^2^)

**Table:**

	*x*	*y*	*z*	*U*_iso_*/*U*_eq_	
S1	0.18342 (2)	0.24406 (4)	0.20047 (2)	0.02336 (9)	
O1	−0.09228 (8)	−0.40177 (12)	0.13728 (8)	0.0385 (3)	
N1	−0.12541 (8)	−0.30589 (14)	0.08755 (8)	0.0284 (3)	
C1	−0.08817 (8)	−0.15360 (15)	0.09366 (8)	0.0233 (3)	
C2	−0.01765 (9)	−0.12471 (16)	0.15533 (9)	0.0281 (3)	
H2	0.0060	−0.2015	0.1935	0.034*	
O2	−0.18878 (8)	−0.32885 (13)	0.03293 (8)	0.0398 (3)	
N2	0.21145 (7)	0.51326 (12)	0.14588 (7)	0.0197 (2)	
C3	0.01769 (9)	0.01841 (16)	0.16020 (9)	0.0281 (3)	
H3	0.0656	0.0402	0.2027	0.034*	
O3	0.08023 (6)	0.58248 (11)	0.07287 (7)	0.0285 (2)	
N3	0.33242 (7)	0.41049 (12)	0.22794 (7)	0.0243 (2)	
C4	−0.01599 (8)	0.13165 (14)	0.10343 (8)	0.0208 (2)	
O4	0.23121 (8)	0.72664 (12)	0.26528 (7)	0.0359 (3)	
C5	−0.08835 (8)	0.09794 (15)	0.04270 (9)	0.0240 (3)	
H5	−0.1128	0.1742	0.0046	0.029*	
O5	0.45456 (6)	0.24066 (12)	0.17337 (7)	0.0299 (2)	
C6	−0.12451 (9)	−0.04434 (16)	0.03746 (9)	0.0263 (3)	
H6	−0.1733	−0.0666	−0.0039	0.032*	
C7	0.02086 (8)	0.28231 (14)	0.10052 (8)	0.0215 (2)	
H7	−0.0160	0.3543	0.0692	0.026*	
C8	0.09867 (8)	0.33479 (14)	0.13522 (8)	0.0196 (2)	
C10	0.25405 (8)	0.39911 (13)	0.19484 (8)	0.0186 (2)	
C24	0.52583 (11)	0.1648 (2)	0.14553 (11)	0.0417 (4)	
H24A	0.5792	0.1890	0.1839	0.063*	
H24B	0.5313	0.1974	0.0877	0.063*	
H24C	0.5159	0.0559	0.1459	0.063*	
C22	0.44054 (8)	0.21301 (15)	0.25472 (9)	0.0248 (3)	
C21	0.48700 (9)	0.11030 (17)	0.30961 (10)	0.0320 (3)	
H21	0.5336	0.0556	0.2922	0.038*	
C20	0.46488 (11)	0.0882 (2)	0.38997 (11)	0.0401 (4)	
H20	0.4965	0.0174	0.4272	0.048*	
C19	0.39771 (11)	0.1670 (2)	0.41689 (11)	0.0400 (4)	
H19	0.3829	0.1502	0.4720	0.048*	
C17	0.37238 (8)	0.29463 (15)	0.28143 (9)	0.0240 (3)	
C9	0.12547 (8)	0.48965 (14)	0.11388 (8)	0.0197 (2)	
C16	0.26397 (9)	0.76094 (15)	0.19277 (9)	0.0252 (3)	
C23	0.23024 (13)	0.8433 (2)	0.32592 (11)	0.0458 (4)	
H23A	0.2899	0.8730	0.3479	0.069*	
H23B	0.2016	0.8074	0.3729	0.069*	
H23C	0.1986	0.9302	0.2989	0.069*	
C15	0.30410 (9)	0.89616 (16)	0.17808 (10)	0.0316 (3)	
H15	0.3118	0.9722	0.2206	0.038*	
C14	0.33285 (10)	0.91874 (18)	0.10035 (12)	0.0383 (4)	
H14	0.3593	1.0118	0.0898	0.046*	
C13	0.32383 (10)	0.80902 (19)	0.03814 (11)	0.0374 (3)	
H13	0.3438	0.8260	−0.0146	0.045*	
C12	0.28481 (9)	0.67286 (16)	0.05420 (9)	0.0278 (3)	
H12	0.2790	0.5954	0.0125	0.033*	
C11	0.25487 (8)	0.65060 (14)	0.13014 (8)	0.0206 (2)	
C18	0.35185 (9)	0.27144 (18)	0.36216 (10)	0.0324 (3)	
H18	0.3061	0.3272	0.3805	0.039*	

### Atomic displacement parameters (Å^2^)

**Table:**

	*U*^11^	*U*^22^	*U*^33^	*U*^12^	*U*^13^	*U*^23^
S1	0.02100 (15)	0.01858 (15)	0.02879 (17)	−0.00265 (11)	−0.00189 (11)	0.00798 (12)
O1	0.0485 (7)	0.0222 (5)	0.0483 (7)	−0.0063 (5)	0.0185 (5)	0.0037 (5)
N1	0.0334 (6)	0.0233 (6)	0.0326 (6)	−0.0093 (5)	0.0178 (5)	−0.0068 (5)
C1	0.0274 (6)	0.0189 (6)	0.0256 (6)	−0.0076 (5)	0.0106 (5)	−0.0033 (5)
C2	0.0321 (7)	0.0236 (7)	0.0280 (7)	−0.0056 (5)	0.0027 (5)	0.0064 (5)
O2	0.0416 (6)	0.0358 (6)	0.0428 (6)	−0.0193 (5)	0.0092 (5)	−0.0119 (5)
N2	0.0196 (5)	0.0146 (5)	0.0239 (5)	−0.0008 (4)	−0.0003 (4)	0.0028 (4)
C3	0.0294 (7)	0.0260 (7)	0.0262 (6)	−0.0074 (5)	−0.0044 (5)	0.0061 (5)
O3	0.0240 (4)	0.0207 (5)	0.0372 (5)	−0.0010 (4)	−0.0066 (4)	0.0082 (4)
N3	0.0198 (5)	0.0196 (5)	0.0324 (6)	0.0004 (4)	0.0005 (4)	0.0051 (4)
C4	0.0205 (5)	0.0201 (6)	0.0218 (6)	−0.0033 (4)	0.0031 (4)	0.0011 (5)
O4	0.0530 (7)	0.0298 (6)	0.0261 (5)	0.0006 (5)	0.0101 (5)	−0.0058 (4)
C5	0.0210 (6)	0.0232 (6)	0.0268 (6)	−0.0017 (5)	0.0002 (5)	0.0019 (5)
O5	0.0278 (5)	0.0295 (5)	0.0319 (5)	0.0047 (4)	0.0031 (4)	0.0023 (4)
C6	0.0230 (6)	0.0275 (7)	0.0276 (6)	−0.0062 (5)	0.0014 (5)	−0.0028 (5)
C7	0.0220 (6)	0.0193 (6)	0.0224 (6)	−0.0016 (4)	0.0008 (4)	0.0027 (5)
C8	0.0211 (5)	0.0171 (6)	0.0200 (5)	0.0000 (4)	0.0008 (4)	0.0024 (4)
C10	0.0199 (5)	0.0154 (5)	0.0206 (6)	−0.0003 (4)	0.0030 (4)	0.0011 (4)
C24	0.0321 (8)	0.0496 (10)	0.0443 (9)	0.0073 (7)	0.0087 (7)	−0.0022 (8)
C22	0.0201 (6)	0.0221 (6)	0.0302 (7)	−0.0014 (5)	−0.0026 (5)	0.0013 (5)
C21	0.0251 (6)	0.0283 (7)	0.0399 (8)	0.0060 (5)	−0.0038 (6)	0.0039 (6)
C20	0.0360 (8)	0.0399 (9)	0.0407 (9)	0.0069 (7)	−0.0065 (6)	0.0145 (7)
C19	0.0365 (8)	0.0484 (10)	0.0342 (8)	0.0019 (7)	0.0019 (6)	0.0147 (7)
C17	0.0179 (5)	0.0201 (6)	0.0322 (7)	−0.0014 (4)	−0.0028 (5)	0.0049 (5)
C9	0.0197 (5)	0.0181 (6)	0.0204 (6)	−0.0013 (4)	0.0002 (4)	0.0005 (4)
C16	0.0245 (6)	0.0209 (6)	0.0287 (6)	0.0022 (5)	−0.0003 (5)	−0.0001 (5)
C23	0.0633 (11)	0.0397 (9)	0.0350 (9)	0.0094 (8)	0.0090 (8)	−0.0132 (7)
C15	0.0308 (7)	0.0183 (6)	0.0430 (8)	−0.0021 (5)	−0.0029 (6)	−0.0033 (6)
C14	0.0310 (7)	0.0261 (7)	0.0564 (10)	−0.0091 (6)	0.0025 (7)	0.0095 (7)
C13	0.0379 (8)	0.0373 (9)	0.0390 (8)	−0.0073 (7)	0.0125 (6)	0.0106 (7)
C12	0.0289 (6)	0.0266 (7)	0.0291 (7)	−0.0011 (5)	0.0076 (5)	0.0032 (5)
C11	0.0195 (5)	0.0167 (6)	0.0246 (6)	−0.0006 (4)	0.0003 (4)	0.0020 (5)
C18	0.0246 (6)	0.0359 (8)	0.0364 (8)	0.0016 (6)	0.0037 (5)	0.0068 (6)

### Geometric parameters (Å, º)

**Table:**

S1—C8	1.7507 (12)	C8—C9	1.4914 (17)
S1—C10	1.7747 (12)	C24—H24A	0.9800
O1—N1	1.2229 (17)	C24—H24B	0.9800
N1—O2	1.2317 (16)	C24—H24C	0.9800
N1—C1	1.4696 (17)	C22—C21	1.3893 (18)
C1—C6	1.3821 (19)	C22—C17	1.4046 (19)
C1—C2	1.3841 (19)	C21—C20	1.386 (2)
C2—C3	1.3833 (19)	C21—H21	0.9500
C2—H2	0.9500	C20—C19	1.381 (3)
N2—C9	1.3783 (15)	C20—H20	0.9500
N2—C10	1.3860 (15)	C19—C18	1.394 (2)
N2—C11	1.4355 (16)	C19—H19	0.9500
C3—C4	1.4001 (18)	C17—C18	1.384 (2)
C3—H3	0.9500	C16—C11	1.3891 (18)
O3—C9	1.2107 (15)	C16—C15	1.3903 (19)
N3—C10	1.2613 (16)	C23—H23A	0.9800
N3—C17	1.4186 (16)	C23—H23B	0.9800
C4—C5	1.4040 (17)	C23—H23C	0.9800
C4—C7	1.4600 (17)	C15—C14	1.391 (2)
O4—C16	1.3636 (18)	C15—H15	0.9500
O4—C23	1.4169 (19)	C14—C13	1.381 (2)
C5—C6	1.3814 (19)	C14—H14	0.9500
C5—H5	0.9500	C13—C12	1.395 (2)
O5—C22	1.3658 (17)	C13—H13	0.9500
O5—C24	1.4265 (19)	C12—C11	1.3724 (19)
C6—H6	0.9500	C12—H12	0.9500
C7—C8	1.3404 (17)	C18—H18	0.9500
C7—H7	0.9500		
			
C8—S1—C10	91.89 (6)	O5—C22—C17	115.42 (11)
O1—N1—O2	123.91 (12)	C21—C22—C17	119.79 (13)
O1—N1—C1	118.25 (12)	C20—C21—C22	119.56 (14)
O2—N1—C1	117.84 (13)	C20—C21—H21	120.2
C6—C1—C2	122.26 (12)	C22—C21—H21	120.2
C6—C1—N1	118.91 (12)	C19—C20—C21	121.28 (14)
C2—C1—N1	118.83 (12)	C19—C20—H20	119.4
C3—C2—C1	118.57 (13)	C21—C20—H20	119.4
C3—C2—H2	120.7	C20—C19—C18	119.12 (15)
C1—C2—H2	120.7	C20—C19—H19	120.4
C9—N2—C10	117.06 (10)	C18—C19—H19	120.4
C9—N2—C11	121.60 (10)	C18—C17—C22	119.63 (12)
C10—N2—C11	121.34 (10)	C18—C17—N3	121.45 (13)
C2—C3—C4	121.06 (12)	C22—C17—N3	118.57 (12)
C2—C3—H3	119.5	O3—C9—N2	123.54 (11)
C4—C3—H3	119.5	O3—C9—C8	126.13 (11)
C10—N3—C17	120.27 (11)	N2—C9—C8	110.31 (10)
C3—C4—C5	118.42 (12)	O4—C16—C11	115.92 (12)
C3—C4—C7	124.55 (11)	O4—C16—C15	124.77 (13)
C5—C4—C7	116.99 (11)	C11—C16—C15	119.31 (13)
C16—O4—C23	117.08 (13)	O4—C23—H23A	109.5
C6—C5—C4	121.06 (12)	O4—C23—H23B	109.5
C6—C5—H5	119.5	H23A—C23—H23B	109.5
C4—C5—H5	119.5	O4—C23—H23C	109.5
C22—O5—C24	116.85 (12)	H23A—C23—H23C	109.5
C5—C6—C1	118.61 (12)	H23B—C23—H23C	109.5
C5—C6—H6	120.7	C16—C15—C14	119.15 (14)
C1—C6—H6	120.7	C16—C15—H15	120.4
C8—C7—C4	130.00 (12)	C14—C15—H15	120.4
C8—C7—H7	115.0	C13—C14—C15	121.52 (14)
C4—C7—H7	115.0	C13—C14—H14	119.2
C7—C8—C9	119.66 (11)	C15—C14—H14	119.2
C7—C8—S1	129.92 (10)	C14—C13—C12	118.78 (14)
C9—C8—S1	110.27 (8)	C14—C13—H13	120.6
N3—C10—N2	121.98 (11)	C12—C13—H13	120.6
N3—C10—S1	127.75 (10)	C11—C12—C13	120.09 (14)
N2—C10—S1	110.27 (8)	C11—C12—H12	120.0
O5—C24—H24A	109.5	C13—C12—H12	120.0
O5—C24—H24B	109.5	C12—C11—C16	121.13 (12)
H24A—C24—H24B	109.5	C12—C11—N2	120.51 (12)
O5—C24—H24C	109.5	C16—C11—N2	118.35 (12)
H24A—C24—H24C	109.5	C17—C18—C19	120.61 (14)
H24B—C24—H24C	109.5	C17—C18—H18	119.7
O5—C22—C21	124.77 (13)	C19—C18—H18	119.7
			
O1—N1—C1—C6	179.51 (12)	O5—C22—C17—C18	178.38 (12)
O2—N1—C1—C6	−1.12 (18)	C21—C22—C17—C18	−0.3 (2)
O1—N1—C1—C2	−0.23 (18)	O5—C22—C17—N3	−8.25 (17)
O2—N1—C1—C2	179.14 (13)	C21—C22—C17—N3	173.07 (12)
C6—C1—C2—C3	−0.4 (2)	C10—N3—C17—C18	−71.58 (18)
N1—C1—C2—C3	179.33 (13)	C10—N3—C17—C22	115.18 (15)
C1—C2—C3—C4	−0.9 (2)	C10—N2—C9—O3	177.23 (12)
C2—C3—C4—C5	1.8 (2)	C11—N2—C9—O3	−2.1 (2)
C2—C3—C4—C7	−175.75 (13)	C10—N2—C9—C8	−4.25 (15)
C3—C4—C5—C6	−1.5 (2)	C11—N2—C9—C8	176.45 (11)
C7—C4—C5—C6	176.26 (13)	C7—C8—C9—O3	7.2 (2)
C4—C5—C6—C1	0.3 (2)	S1—C8—C9—O3	−176.77 (12)
C2—C1—C6—C5	0.7 (2)	C7—C8—C9—N2	−171.26 (12)
N1—C1—C6—C5	−179.01 (12)	S1—C8—C9—N2	4.76 (13)
C3—C4—C7—C8	14.9 (2)	C23—O4—C16—C11	−172.66 (13)
C5—C4—C7—C8	−162.70 (14)	C23—O4—C16—C15	6.8 (2)
C4—C7—C8—C9	172.27 (13)	O4—C16—C15—C14	−178.36 (13)
C4—C7—C8—S1	−2.9 (2)	C11—C16—C15—C14	1.1 (2)
C10—S1—C8—C7	172.24 (13)	C16—C15—C14—C13	−1.2 (2)
C10—S1—C8—C9	−3.25 (9)	C15—C14—C13—C12	0.1 (2)
C17—N3—C10—N2	176.56 (12)	C14—C13—C12—C11	1.1 (2)
C17—N3—C10—S1	−4.5 (2)	C13—C12—C11—C16	−1.1 (2)
C9—N2—C10—N3	−179.07 (12)	C13—C12—C11—N2	177.44 (13)
C11—N2—C10—N3	0.23 (19)	O4—C16—C11—C12	179.55 (12)
C9—N2—C10—S1	1.80 (14)	C15—C16—C11—C12	0.03 (19)
C11—N2—C10—S1	−178.90 (9)	O4—C16—C11—N2	0.94 (17)
C8—S1—C10—N3	−178.06 (13)	C15—C16—C11—N2	−178.58 (12)
C8—S1—C10—N2	1.01 (9)	C9—N2—C11—C12	−79.97 (16)
C24—O5—C22—C21	−4.2 (2)	C10—N2—C11—C12	100.76 (15)
C24—O5—C22—C17	177.21 (13)	C9—N2—C11—C16	98.65 (14)
O5—C22—C21—C20	−177.81 (14)	C10—N2—C11—C16	−80.62 (15)
C17—C22—C21—C20	0.7 (2)	C22—C17—C18—C19	−0.5 (2)
C22—C21—C20—C19	−0.4 (2)	N3—C17—C18—C19	−173.70 (14)
C21—C20—C19—C18	−0.5 (3)	C20—C19—C18—C17	0.9 (2)

### Hydrogen-bond geometry (Å, º)

**Table:**

*D*—H···*A*	*D*—H	H···*A*	*D*···*A*	*D*—H···*A*
C3—H3···S1	0.95	2.58	3.2594 (14)	128
C3—H3···O1^i^	0.95	2.57	3.3320 (18)	138
C5—H5···O3^ii^	0.95	2.58	3.3938 (17)	145
C7—H7···O3^ii^	0.95	2.40	3.1982 (15)	142
C21—H21···N3^iii^	0.95	2.52	3.4576 (19)	170
C23—H23*C*···O1^iv^	0.98	2.55	3.238 (2)	127

Symmetry codes: (i) −*x*, *y*+1/2, −*z*+1/2; (ii) −*x*, −*y*+1, −*z*; (iii) −*x*+1, *y*−1/2, −*z*+1/2; (iv) −*x*, *y*+3/2, −*z*+1/2.
