# Supplementary material for: Crystal and mol­ecular structure of (2Z,5Z)-3-(2-meth­oxy­phen­yl)-2-[(2-meth­oxy­phen­yl)imino]-5-(4-nitro­benzyl­idene)thia­zolidin-4-one
Source: Acta Crystallogr E Crystallogr Commun. 2017 Mar 14;73(Pt 4):511–4. doi: 10.1107/S2056989017003218 (PMC5382610; doi:10.1107/S2056989017003218)
Supplement: Supplementary file 4 [file e-73-00511-sup3.docx]

**Table S1** Geometric parameters by theoretical calculations (Å, °)

| Bond lengths |  | Bond angles |  | Dihedral angles |  |
| --- | --- | --- | --- | --- | --- |
| S1−C8 | 1.762 | S1−C8−C7 | 126.83 | C1–C2–C3–C4 | -0.08 |
| S1−C10 | 1.816 | S1−C8−C9 | 109.74 | C2–C3–C4–C5 | 0.21 |
| O1−N1 | 1.231 | S1−C10−N2 | 110.93 | C2–C3–C4–C7 | -179.79 |
| O2−N1 | 1.231 | S1−C10−N3 | 130.23 | C2–C1–N1–O1 | -0.10 |
| O3−C9 | 1.215 | O1−N1−O2 | 124.63 | C2–C1–N1–O2 | 179.91 |
| O4−C16 | 1.359 | O1−N1−C1 | 117.65 | C3–C4–C5–C6 | -0.19 |
| O4−C23 | 1.420 | O2−N1−C1 | 117.70 | C3–C4–C7–C8 | 178.06 |
| O5−C22 | 1.362 | O3−C9−C8 | 125.29 | C3–C2–C1–N1 | 179.98 |
| O5−C24 | 1.418 | O3−C9−N2 | 124.29 | C4–C5–C6–C1 | 0.03 |
| N1−C1 | 1.468 | O4−C16−C11 | 115.95 | C4–C7–C8–C9 | -179.64 |
| N2−C9 | 1.394 | O4−C16−C15 | 125.10 | C4–C7–C8–S1 | -0.35 |
| N2−C10 | 1.393 | O5−C22−C17 | 115.52 | C5–C4–C7–C8 | -1.94 |
| N2−C11 | 1.434 | O5−C22−C21 | 124.80 | C5–C6–C1–C2 | 0.10 |
| N3−C10 | 1.263 | N1−C1−C2 | 119.12 | C5–C6–C1–N1 | -179.96 |
| N3−C17 | 1.406 | N1−C1−C6 | 119.13 | C6–C1–C2–C3 | -0.07 |
| C1−C2 | 1.394 | N2−C9−C8 | 110.40 | C6–C5–C4–C7 | 179.81 |
| C1−C6 | 1.394 | N2−C10−N3 | 123.39 | C6–C1–N1–O1 | 179.96 |
| C2−C3 | 1.386 | N2−C11−C12 | 120.52 | C6–C1–N1–O2 | -0.02 |
| C3−C4 | 1.413 | N2−C11−C16 | 118.69 | C7–C8–C9–N2 | -178.66 |
| C4−C5 | 1.412 | N3−C17−C18 | 121.86 | C7–C8–C9–O3 | 0.81 |
| C5−C6 | 1.388 | N3−C17−C22 | 118.82 | C7–C8–S1–C10 | 179.49 |
| C4−C7 | 1.455 | C1−C2−C3 | 118.57 | C8–C9–N2–C10 | -1.906 |
| C7−C8 | 1.351 | C1−C6−C5 | 119.09 | C8–C9–N2–C11 | -175.63 |
| C8−C9 | 1.498 | C2−C3−C4 | 121.61 | C8–S1–C10–N2 | 0.16 |
| C11−C12 | 1.388 | C2−C1−C6 | 121.73 | C8–S1–C10–N3 | -178.46 |
| C12−C13 | 1.395 | C3−C4−C5 | 117.94 | C9–C8–S1–C10 | -1.17 |
| C13−C14 | 1.392 | C4−C5−C6 | 121.03 | C9–N2–C10–S1 | 1.01 |
| C14−C15 | 1.397 | C3−C4−C7 | 117.37 | C9–N2–C10–N3 | 179.70 |
| C15−C16 | 1.398 | C5−C4−C7 | 124.67 | O3–C9–N2–C10 | 178.61 |
| C16−C11 | 1.407 | C4−C7−C8 | 131.56 | O3–C9–N2–C11 | 4.88 |
| C17−C18 | 1.398 | C7−C8−C9 | 118.82 | O3–C9–C8–S1 | -178.60 |
| C18−C19 | 1.397 | C8−S1−C10 | 91.49 | N2–C9–C8–S1 | 1.91 |
| C19−C20 | 1.390 | C9−N2−C10 | 117.38 | N2–C10–N3–C17 | 177.12 |
| C20−C21 | 1.399 | C9−N2−C11 | 121.14 | N2–C11–C12–C13 | 179.16 |
| C21−C22 | 1.396 | C10−N2−C11 | 121.16 | N2–C11–C16–C15 | -179.27 |
| C22−C17 | 1.416 | C10−N3−C17 | 122.06 | N2–C11–C16–O4 | 0.65 |
|  |  | C11−C12−C13 | 120.17 | C10–N2–C11–C12 | 96.25 |
|  |  | C12−C13−C14 | 119.25 | C10–N2–C11–C16 | -84.61 |
|  |  | C13−C14−C15 | 121.03 | C10–N3–C17–C18 | -67.40 |
|  |  | C14−C15−C16 | 119.82 | C10–N3–C17–C22 | 117.72 |
|  |  | C15−C16−C11 | 118.93 | S1–C10–N2–C11 | 174.74 |
|  |  | C17−C18−C19 | 120.98 | S1–C10–N3–C17 | -4.41 |
|  |  | C18−C19−C20 | 119.55 | C11–C12–C13–C14 | 0.05 |
|  |  | C19−C20−C21 | 120.39 | C11–C16–O4–C23 | -179.92 |
|  |  | C20−C21−C22 | 120.28 | C11–C16–C15–C14 | 0.14 |
|  |  | C21−C22−C17 | 119.66 | C12–C13–C14–C15 | -0.04 |
|  |  | C22−C17−C18 | 119.11 | C12–C11–C16–O4 | 179.78 |
|  |  | C16−O4−C23 | 118.27 | C12–C11–C16–C15 | -0.14 |
|  |  | C22−O5−C24 | 118.11 | C13–C14–C15–C16 | -0.04 |
|  |  |  |  | C13–C12–C11–C16 | 0.04 |
|  |  |  |  | C14–C15–C16–O4 | -179.78 |
|  |  |  |  | C15–C16–O4–C23 | 0.00 |
|  |  |  |  | N3–C17–C18–C19 | -175.51 |
|  |  |  |  | N3–C17–C22–C21 | 175.74 |
|  |  |  |  | N3–C17–C22–O5 | -5.17 |
|  |  |  |  | C17–C18–C19–C20 | 0.28 |
|  |  |  |  | C17–C22–C21–C20 | -0.44 |
|  |  |  |  | C17–C22–O5–C24 | 178.27 |
|  |  |  |  | C18–C19–C20–C21 | 0.02 |
|  |  |  |  | C18–C17–C22–C21 | 0.73 |
|  |  |  |  | C18–C17–C22–O5 | 179.81 |
|  |  |  |  | C19–C20–C21–C22 | 0.05 |
|  |  |  |  | C20–C21–C22–O5 | -179.42 |
|  |  |  |  | C21–C22–O5–C24 | -2.69 |
