# Supplementary material for: Crystal and mol­ecular structure of (2Z,5Z)-3-(2-meth­oxy­phen­yl)-2-[(2-meth­oxy­phen­yl)imino]-5-(4-nitro­benzyl­idene)thia­zolidin-4-one
Source: Acta Crystallogr E Crystallogr Commun. 2017 Mar 14;73(Pt 4):511–4. doi: 10.1107/S2056989017003218 (PMC5382610; doi:10.1107/S2056989017003218)
Supplement: Supplementary file 5 [file e-73-00511-sup3.pdf]

Table S1 Geometric parameters by theoretical calculations (Å, °)

Bond lengths		Bond angles		Dihedral angles	
S1–C8	1.762	S1–C8–C7	126.83	C1–C2–C3–C4	-0.08
S1–C10	1.816	S1–C8–C9	109.74	C2–C3–C4–C5	0.21
O1–N1	1.231	S1–C10–N2	110.93	C2–C3–C4–C7	-179.79
O2–N1	1.231	S1–C10–N3	130.23	C2–C1–N1–O1	-0.10
O3–C9	1.215	O1–N1–O2	124.63	C2–C1–N1–O2	179.91
O4–C16	1.359	O1–N1–C1	117.65	C3–C4–C5–C6	-0.19
O4–C23	1.420	O2–N1–C1	117.70	C3–C4–C7–C8	178.06
O5–C22	1.362	O3–C9–C8	125.29	C3–C2–C1–N1	179.98
O5–C24	1.418	O3–C9–N2	124.29	C4–C5–C6–C1	0.03
N1–C1	1.468	O4–C16–C11	115.95	C4–C7–C8–C9	-179.64
N2–C9	1.394	O4–C16–C15	125.10	C4–C7–C8–S1	-0.35
N2–C10	1.393	O5–C22–C17	115.52	C5–C4–C7–C8	-1.94
N2–C11	1.434	O5–C22–C21	124.80	C5–C6–C1–C2	0.10
N3–C10	1.263	N1–C1–C2	119.12	C5–C6–C1–N1	-179.96
N3–C17	1.406	N1–C1–C6	119.13	C6–C1–C2–C3	-0.07
C1–C2	1.394	N2–C9–C8	110.40	C6–C5–C4–C7	179.81
C1–C6	1.394	N2–C10–N3	123.39	C6–C1–N1–O1	179.96
C2–C3	1.386	N2–C11–C12	120.52	C6–C1–N1–O2	-0.02
C3–C4	1.413	N2–C11–C16	118.69	C7–C8–C9–N2	-178.66
C4–C5	1.412	N3–C17–C18	121.86	C7–C8–C9–O3	0.81
C5–C6	1.388	N3–C17–C22	118.82	C7–C8–S1–C10	179.49
C4–C7	1.455	C1–C2–C3	118.57	C8–C9–N2–C10	-1.906
C7–C8	1.351	C1–C6–C5	119.09	C8–C9–N2–C11	-175.63
C8–C9	1.498	C2–C3–C4	121.61	C8–S1–C10–N2	0.16
C11–C12	1.388	C2–C1–C6	121.73	C8–S1–C10–N3	-178.46
C12–C13	1.395	C3–C4–C5	117.94	C9–C8–S1–C10	-1.17
C13–C14	1.392	C4–C5–C6	121.03	C9–N2–C10–S1	1.01
C14–C15	1.397	C3–C4–C7	117.37	C9–N2–C10–N3	179.70
C15–C16	1.398	C5–C4–C7	124.67	O3–C9–N2–C10	178.61
C16–C11	1.407	C4–C7–C8	131.56	O3–C9–N2–C11	4.88
C17–C18	1.398	C7–C8–C9	118.82	O3–C9–C8–S1	-178.60
C18–C19	1.397	C8–S1–C10	91.49	N2–C9–C8–S1	1.91
C19–C20	1.390	C9–N2–C10	117.38	N2–C10–N3–C17	177.12
C20–C21	1.399	C9–N2–C11	121.14	N2–C11–C12–C13	179.16
C21–C22	1.396	C10–N2–C11	121.16	N2–C11–C16–C15	-179.27
C22–C17	1.416	C10–N3–C17	122.06	N2–C11–C16–O4	0.65
		C11–C12–C13	120.17	C10–N2–C11–C12	96.25
		C12–C13–C14	119.25	C10–N2–C11–C16	-84.61
		C13–C14–C15	121.03	C10–N3–C17–C18	-67.40
		C14–C15–C16	119.82	C10–N3–C17–C22	117.72
		C15–C16–C11	118.93	S1–C10–N2–C11	174.74
		C17–C18–C19	120.98	S1–C10–N3–C17	-4.41
		C18–C19–C20	119.55	C11–C12–C13–C14	0.05

C19–C20–C21	120.39	C11–C16–O4–C23	-179.92
C20–C21–C22	120.28	C11–C16–C15–C14	0.14
C21–C22–C17	119.66	C12–C13–C14–C15	-0.04
C22–C17–C18	119.11	C12–C11–C16–O4	179.78
C16–O4–C23	118.27	C12–C11–C16–C15	-0.14
C22–O5–C24	118.11	C13–C14–C15–C16	-0.04
		C13–C12–C11–C16	0.04
		C14–C15–C16–O4	-179.78
		C15–C16–O4–C23	0.00
		N3–C17–C18–C19	-175.51
		N3–C17–C22–C21	175.74
		N3–C17–C22–O5	-5.17
		C17–C18–C19–C20	0.28
		C17–C22–C21–C20	-0.44
		C17–C22–O5–C24	178.27
		C18–C19–C20–C21	0.02
		C18–C17–C22–C21	0.73
		C18–C17–C22–O5	179.81
		C19–C20–C21–C22	0.05
		C20–C21–C22–O5	-179.42
		C21–C22–O5–C24	-2.69
